# Association between platelet-to-white blood cell ratio and short-term prognosis in patients with acute myocardial infarction: a retrospective cohort study

**DOI:** 10.3389/fcvm.2026.1935579

**Published:** 2026-10-27

**Authors:** Zelin Kang, Yongxin Liao, Chong Zheng, Zhaokang Li, Gengzhen Yao, Xu Zou

**Affiliations:** 1 The Second Clinical College of Guangzhou University of Chinese Medicine, Guangzhou, China; 2 Department of Dermatology, Xingtan Branch of the Eighth Affiliated Hospital of Southern Medical University, Foshan, China; 3 Department of Cardiology, Guangdong Provincial Hospital of Chinese Medicine, Guangzhou, China

**Keywords:** platelet-to-white blood cell ratio, acute myocardial infarction, short-term prognosis, MIMIC-IV database, dual cohort validation

## Abstract

**Background:**

Clinical evidence regarding the prognostic value of the platelet-to-white blood cell ratio (PWR) for short-term outcomes in patients with acute myocardial infarction (AMI) remains limited, particularly its independent predictive role for in-hospital and ICU all-cause mortality, which lacks validation in large-scale, multi-center settings. This study investigated the association between PWR and in-hospital and ICU mortality in AMI patients, aiming to evaluate its impact on short-term prognosis in this population.

**Methods:**

This retrospective cohort study included 2,381 AMI patients from the MIMIC-IV database as the derivation cohort, with validation performed in 7,054 AMI patients from the multi-center eICU-CRD database and 252 AMI patients from a single-center internal cohort. Restricted cubic spline and Kaplan–Meier analyses were used to assess nonlinearity and survival differences. Multivariable logistic regression examined the independent association between PWR and in-hospital and ICU mortality. Decision curve analysis assessed the clinical utility of PWR. Subgroup analyses, Cox regression, Fine-Gray competing-risk models, and mediation analyses were conducted for robustness assessment and mechanistic exploration.

**Results:**

In the derivation cohort of this study, 316 patients (13.3%) died during hospitalization and 237 (10.0%) died during ICU stay. Restricted cubic spline analysis demonstrated a consistent linear relationship between PWR and the risks of in-hospital and ICU mortality. Multivariable logistic regression revealed a significant inverse association between PWR and in-hospital mortality (OR = 0.976, 95% CI: 0.960–0.991, P = 0.003) as well as ICU mortality (OR = 0.977, 95% CI: 0.959–0.994, P = 0.012). The robustness of this inverse association was further supported by Cox regression and Fine-Gray competing-risk models. The results were consistently reproduced in both the external eICU-CRD validation cohort and the single-center internal validation cohort. Additionally, Kaplan–Meier curves showed that patients in the higher PWR group had significantly better cumulative survival than those in the lower PWR group. Decision curve analysis further showed that PWR provided some net benefit at clinically relevant thresholds for both outcomes, supporting its clinical utility. In subgroup analyses, the inverse association between PWR and short-term mortality remained directionally consistent across all subgroups, with no significant interactions after Benjamini–Hochberg FDR correction. Mediation analysis indicated that SAPS II score and vasopressor use mediated 25.9% and 18.6% of the protective effect of PWR, respectively.

**Conclusions:**

PWR is a simple, low-cost composite marker derived from routine complete blood counts. It is significantly and inversely associated with short-term in-hospital and ICU mortality in AMI patients. Our findings suggest that PWR may serve as a convenient and inexpensive adjunctive tool for early risk stratification and identification of high-risk individuals. However, its clinical application warrants further validation in larger, multi-center prospective studies.

## GRAPHICAL ABSTRACT

**Figure:**
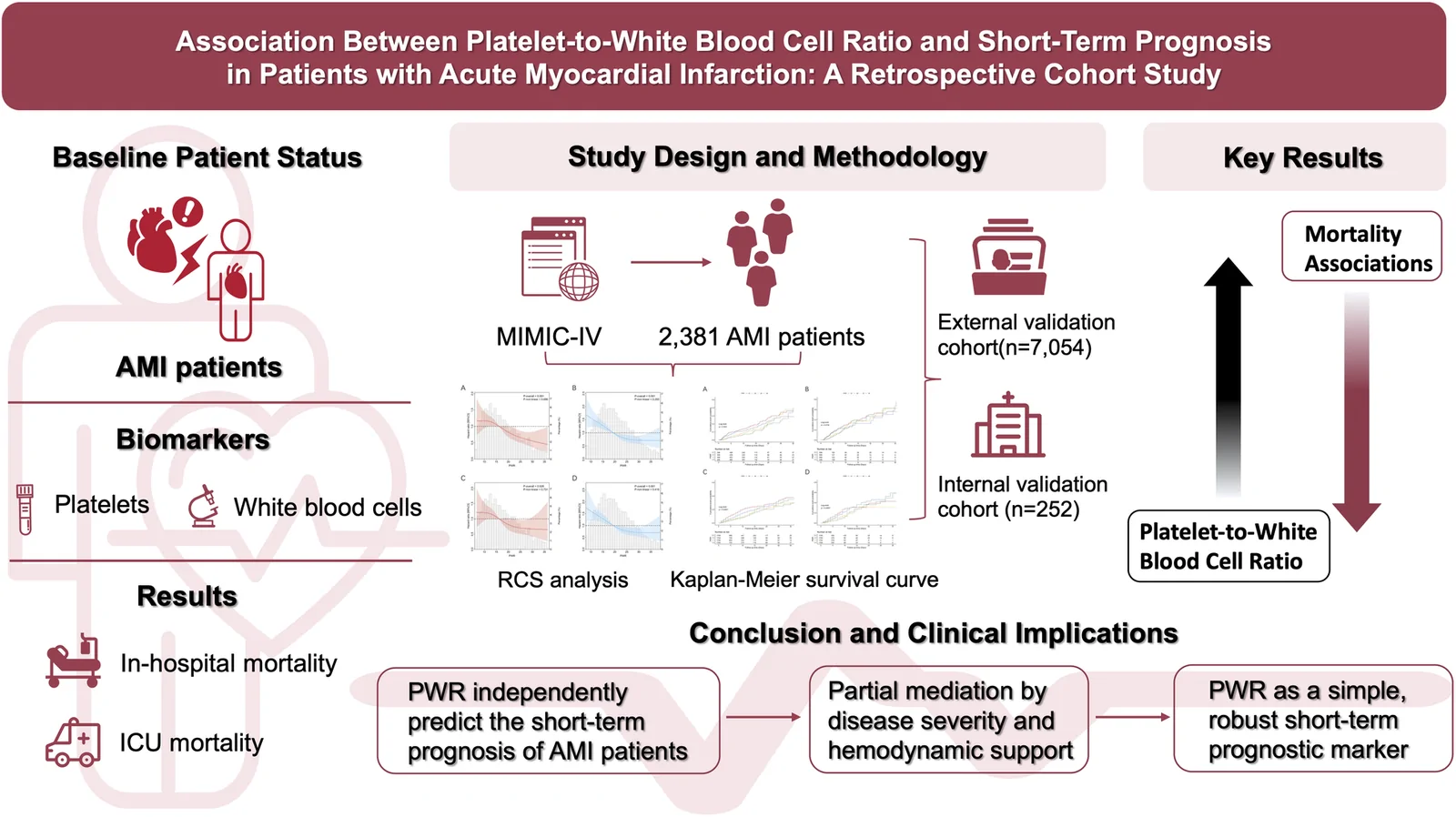
Association between platelet-to-white blood cell ratio (PWR) and short-term prognosis in patients with acute myocardial infarction (AMI): a retrospective cohort study. Higher PWR was independently associated with lower in-hospital and ICU mortality, suggesting that PWR may serve as a simple short-term prognostic marker.

## 1 Introduction

Acute myocardial infarction (AMI) remains a leading cause of cardiovascular death worldwide (1, 2). Even after receiving percutaneous coronary intervention (PCI) and pharmacological therapy, patients with AMI still face a substantial risk of short-term mortality (3), particularly those with multiple comorbidities, in whom in-hospital and intensive care unit (ICU) mortality rates remain discouragingly high (4, 5). Therefore, early identification of high-risk patients and accurate risk stratification are critical to improving outcomes in AMI.

The pathophysiological core of AMI is rupture of coronary atherosclerotic plaques with subsequent thrombosis, a process involving crosstalk between inflammation and platelet activation (6, 7). Although single inflammatory markers such as C-reactive protein (CRP) have been associated with cardiovascular events, a single biomarker cannot adequately reflect the complex state of the inflammation-coagulation network (8, 9). In recent years, composite inflammatory indices, including the neutrophil-to-lymphocyte ratio (NLR) and platelet-to-lymphocyte ratio (PLR), have been extensively investigated and have demonstrated superior predictive performance over single markers (10–12). The platelet-to-white blood cell ratio (PWR), which integrates platelet count (reflecting a prothrombotic state) and white blood cell count (reflecting systemic inflammation), simultaneously captures both platelet function and immune response information. Theoretically, PWR can concurrently assess the two major pathways of inflammation and thrombosis, making it a potential novel biomarker for prognostic evaluation in AMI patients (12–14).

Previous studies have suggested an association between PWR and the severity of coronary artery disease as well as short-term prognosis in patients with heart failure (14, 15). However, the independent predictive value of PWR for in-hospital all-cause mortality and ICU mortality specifically in the AMI population has not been systematically validated using large-scale, multi-center data. Moreover, most existing studies rely on a single database without external validation, limiting the generalizability of their findings (13, 16, 17).

To address this gap, we conducted a retrospective analysis based on the Medical Information Mart for Intensive Care IV (MIMIC-IV) large public database to evaluate the association between PWR and in-hospital and ICU short-term mortality in AMI patients. To enhance the generalizability and robustness of our results, we performed dual external validation using the multi-center eICU Collaborative Research Database (eICU) and an internal single-center cohort. Furthermore, we rigorously controlled for multiple confounders and employed analytical approaches including multivariable logistic regression, subgroup analyses, Fine-Gray subdistribution hazard models, and mediation analyses, aiming to provide evidence-based support for the clinical application of PWR in the short-term prognostic assessment of AMI patients.

## 2 Methods

### 2.1 Data sources

Data were derived from three independent databases. The primary derivation cohort came from the MIMIC-IV (version 3.1) public critical care database, which includes patients admitted to the Beth Israel Deaconess Medical Center (Boston, MA, USA) between 2008 and 2019, with >360,000 distinct individuals and >90,000 ICU admissions (18). One validation cohort was sourced from the multi-center eICU-CRD (version 2.0), which collected electronic health records from 208 U.S. hospitals during 2014–2015, covering >200,000 ICU stays (19). Both public databases are de-identified, so informed consent and ethical approval were waived. The other validation cohort was a single-center retrospective internal cohort from Guangdong Provincial Hospital of Traditional Chinese Medicine, with data collected from October 2022 to October 2024. Baseline characteristics, laboratory tests, comorbidities, and outcomes were extracted from the hospital's electronic medical record system. This study was approved by the Ethics Committee of Guangdong Provincial Hospital of Traditional Chinese Medicine (approval no. ZE2026-075), and written informed consent was waived due to the retrospective design.

### 2.2 Study population

We included first-time ICU admissions diagnosed with AMI, defined by ICD-9 and ICD-10 codes, covering both STEMI and NSTEMI. Exclusion criteria: (1) age <18 years; (2) non-first ICU admission; (3) ICU length of stay <24 h; or (4) missing WBC or PLT count on the first ICU day. The selection process is shown in Figure 1.

**Figure 1:**
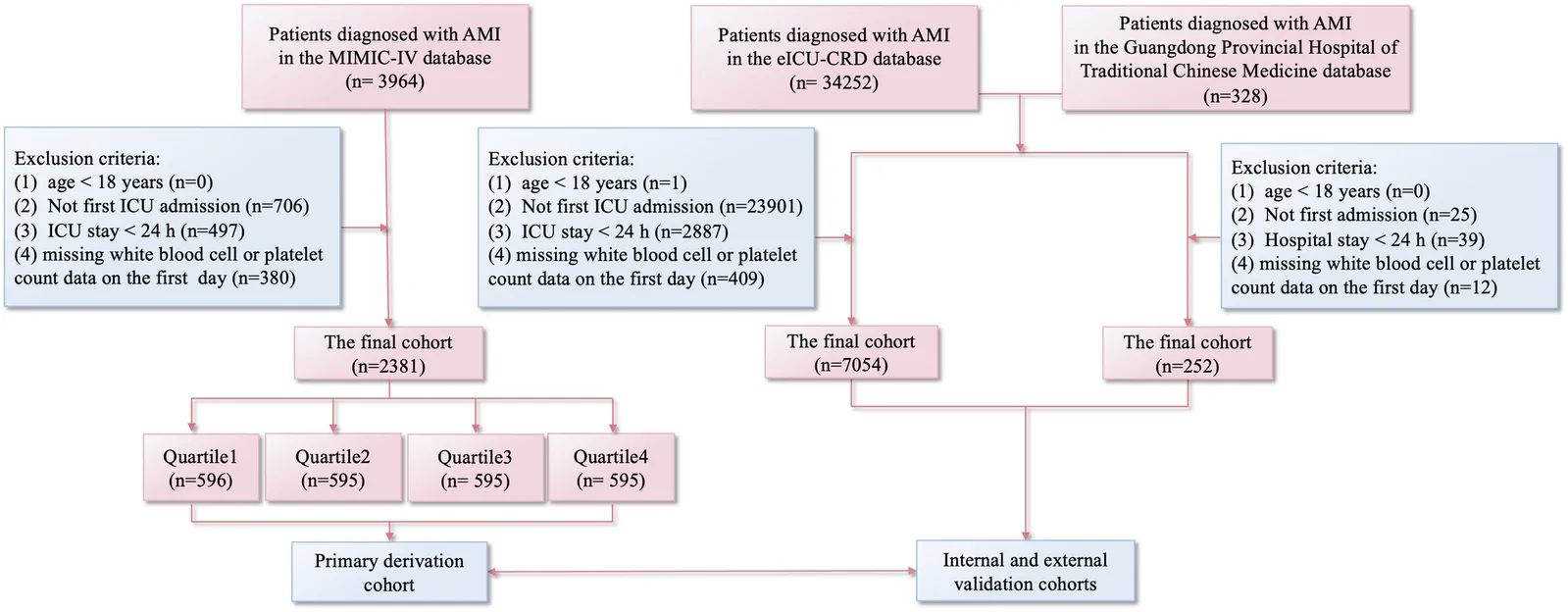
The flow chart of the study participants. AMI, acute myocardial infarction; MIMIC, Medical Information Mart for Intensive Care; eICU-CRD, eICU Collaborative Research Database.

### 2.3 Data extraction

Data were extracted using SQL with PostgreSQL (version 17.1). For the final cohorts, we retrieved: (1) age and sex; (2) comorbidities via ICD codes; (3) first PLT and WBC within 24 h of ICU admission; (4) other laboratory parameters within 24 h; (5) first-day SOFA, APS III, OASIS, and SAPS II; (6) treatments potentially affecting prognosis, including invasive mechanical ventilation, CRRT, vasoactive drugs (dobutamine, dopamine, epinephrine, norepinephrine, milrinone, vasopressin), and statin use. Covariates with more than 20% missing data were excluded from the analysis. For covariates with less than 20% missing data, multiple imputation was performed using the mice package in R. We applied multiple imputation by chained equations (MICE), using classification and regression trees (CART) as the imputation method, to generate 10 imputed datasets (m = 10). All analyses were performed separately within each imputed dataset, and estimates were subsequently pooled using Rubin's rules.

### 2.4 Study variables

PWR was calculated as PLT (K/*μ*L)/WBC (K/μL), using the first recorded values after ICU admission. PWR was analyzed both as a continuous variable and as a categorical variable (quartiles: Q1–Q4, with Q1 as reference).

### 2.5 Primary outcomes

The primary outcomes were all-cause in-hospital mortality and all-cause ICU mortality.

### 2.6 Statistical analysis

Analyses used SPSS 27.0 and R 4.4.1. Continuous variables were expressed as mean ± SD or median (IQR), compared by t-test or Mann–Whitney *U* test; categorical variables as frequencies (percentages), compared by *χ*^2^ or Fisher's exact test. To improve the clinical interpretability and cross-study comparability of effect estimates, we report both the odds ratio (OR) per 1-unit increase in PWR and the per-IQR OR. The IQR for each cohort was calculated from the actual distribution of PWR within that cohort (IQR = Q3−Q1). For continuous PWR fitted as a linear term, the per-IQR OR was calculated as exp (β × IQR), where β is the log-scale coefficient per 1-unit increase in PWR. The 95% CI was obtained by exponentiating (β ± 1.96 × SE) × IQR; *P* values were unchanged. The same approach was applied to HR and sHR by replacing OR with HR or sHR, as appropriate.

To explore nonlinearity, restricted cubic splines (RCS) were fitted with knots at the 5th, 35th, 65th, and 95th percentiles of PWR, trimming extremes below the 5th and above the 95th percentiles. Nonlinearity was tested by Wald test. Kaplan–Meier curves stratified by PWR quartiles were compared using log-rank test.

Multivariable logistic regression was used to assess the independent association of PWR with mortality. Three models were built: Model 1—unadjusted; Model 2—adjusted for age and sex; Model 3—adjusted for creatinine, hemoglobin, calcium, potassium, hypertension, hyperlipidemia, diabetes, atrial fibrillation, congestive heart failure, peripheral vascular disease, cerebrovascular disease, COPD, statin use, aspirin use, clopidogrel use, CRRT, SOFA, and APS III. Multicollinearity was checked by Spearman's correlation and the square root of VIF.

Subgroup analyses were performed stratified by sex, age (>65 vs. ≤ 65 years), hypertension, diabetes, congestive heart failure, atrial fibrillation, COPD, end-stage renal disease, acute renal failure, and sepsis. Interactions were tested by likelihood ratio tests. To mitigate type I error inflation arising from multiple comparisons, the Benjamini-Hochberg procedure was applied to control the false discovery rate (FDR) for interaction tests across all prespecified subgroup variables. An FDR-adjusted *P* < 0.05 was considered statistically significant for interaction. Both unadjusted and FDR-adjusted *P* values for interactions were reported.

Sensitivity analyses included Cox proportional hazards regression and Fine-Gray subdistribution hazard models, with PWR as continuous, for in-hospital, ICU, 180-day, and 365-day mortality. To account for competing risks (alive discharge/transfer), Fine-Gray models treated in-hospital death (or ICU death) as the event and alive discharge (or alive transfer) as the competing event, with time defined as hospital or ICU length of stay, reporting subdistribution hazard ratios (sHR) with 95% CIs. All sensitivity models were adjusted for the same covariates as Model 3. Analyses used R with the *cmprsk* package. To assess the calibration of the PWR, we separately calculated the predicted probabilities of in-hospital and ICU all-cause mortality and evaluated calibration using the Brier score, calibration slope, and calibration intercept. Decision curve analysis (DCA) was performed to assess the clinical value of the model by quantifying net benefit across a range of threshold probabilities (20). Predicted probabilities were generated via repeated 10-fold cross-validation (100 repetitions). The 95% confidence interval (CI) for the difference in net benefit was estimated using 1,000 bootstrap resamples.

For external validation (eICU-CRD) and internal validation (Guangdong hospital), we repeated multivariable logistic regression with Models 1–3, entering PWR both continuously (per unit) and by quartiles (Q1 reference), and tested linear trend by including the median of each quartile as a continuous variable. We calculated the E-value (the minimum risk ratio that an unmeasured confounder would need to have with both exposure and outcome to fully explain away the observed association) based on the point estimate, to assess robustness to unmeasured confounding.

To quantify mediation pathways (disease severity and circulatory failure), we performed mediation analyses using SAPS II and vasoactive drug use as mediators, adjusted for age and sex, with bootstrap (1,000 resamples) to estimate the average causal mediation effect (ACME), average direct effect (ADE), total effect, and proportion mediated. A two-sided *P* < 0.05 was considered significant.

## 3 Results

### 3.1 Baseline characteristics

The primary cohort ultimately included 2,381 AMI patients from the MIMIC-IV database, and their demographic and clinical characteristics are summarized in Table 1. The median age was 70.44 (61.53, 78.46) years, and 1,631 (68.5%) were male. In-hospital mortality was 13.3% (316 cases), ICU mortality was 10.0% (237 cases), and 28-day mortality was 14.7% (349 cases). The median PWR was 16.20 (11.27, 22.90). Patients were divided into Q1 (PWR < 11.27), Q2 (11.27 ≤ PWR < 16.20), Q3 (16.20 ≤ PWR < 22.90), and Q4 (PWR ≥ 22.90). Compared with the lowest quartile (Q1), patients in the highest quartile (Q4) had lower heart rates, lower proportions of vasoactive drug use (e.g., epinephrine, norepinephrine, vasopressin), lower WBC counts, and lower APS III, SOFA, and SAPS II scores (all *P* < 0.001). Furthermore, in the MIMIC-IV cohort, in-hospital mortality, ICU mortality, and 28-day mortality all showed a gradual decreasing trend from Q1 to Q4 (all *P* < 0.05). Notably, 90-day mortality also showed a decreasing trend that was borderline significant (*P* for trend = 0.050), whereas 180-day and 365-day mortality did not differ significantly across groups (*P* > 0.05), suggesting that PWR may predominantly serve as a predictor of short-term prognosis.

**Table 1:** Characteristics and outcomes of participants according to the PWR quartiles^a^.

Variables	Level	Overall (*n* = 2381)	Q1 (*n* = 596)	Q2 (*n* = 595)	Q3 (*n* = 595)	Q4 (*n* = 595)	p
			PWR < 11.27	11.27 ≤ PWR ＜ 16.20	16.20 ≤ PWR ＜ 22.90	PWR ≥ 22.90	
**Demographic variables**							
Age, years		70.44 [61.53, 78.46]	70.09 [61.55, 78.04]	70.30 [61.28, 79.03]	71.27 [62.06, 78.96]	70.02 [61.38, 77.76]	0.456
Male, n(%)		1631 (68.5)	442 (74.2)	415 (69.7)	414 (69.6)	360 (60.5)	<0.001
Race, n(%)	White	1269 (57.5)	346 (58.1)	339 (57.0)	339 (57.0)	345 (58.0)	0.051
	Black	171 (7.2)	39 (6.5)	30 (5.0)	44 (7.4)	58 (9.7)	
	Others	841 (35.3)	211 (35.4)	226 (38.0)	212 (35.6)	192 (32.3)	
Marital status, n(%)	Divorced	162 (6.8)	41 (6.9)	37 (6.2)	43 (7.2)	41 (6.9)	0.006
	Married	1077 (45.2)	279 (46.8)	263 (44.2)	256 (43.0)	279 (46.9)	
	Single	455 (19.1)	106 (17.8)	103 (17.3)	112 (18.8)	134 (22.5)	
	Widowed	223 (9.4)	42 (7.0)	55 (9.2)	68 (11.4)	58 (9.7)	
	Others	464 (19.5)	128 (21.5)	137 (23.0)	116 (19.5)	83 (13.9)	
Weight, kg		81.00 [69.50, 95.00]	84.15 [70.15, 96.03]	81.60 [70.55, 96.40]	81.00 [70.00, 96.20]	78.20 [65.65, 91.25]	<0.001
**Vital signs**							
Temperature, °C		36.61 [36.39, 36.89]	36.56 [36.33, 36.89]	36.56 [36.39, 36.89]	36.67 [36.39, 36.89]	36.67 [36.44, 36.89]	0.05
Heart rate, bpm		82.00 [74.00, 94.00]	84.00 [78.00, 95.25]	83.00 [75.00, 96.00]	81.00 [72.00, 93.00]	80.00 [71.00, 91.00]	<0.001
Respiratory rate, breaths/min		18.00 [15.50, 22.00]	18.00 [16.00, 22.00]	18.00 [16.00, 22.00]	18.00 [16.00, 22.00]	18.00 [15.00, 21.00]	0.068
Systolic BP, mmHg		117.00 [104.00, 133.00]	113.00 [102.00, 129.00]	116.00 [102.00, 130.00]	121.00 [106.00, 136.75]	119.00 [106.00, 134.00]	<0.001
Diastolic BP, mmHg		65.00 [54.00, 77.00]	61.00 [53.00, 73.00]	63.00 [54.00, 75.00]	69.00 [56.00, 79.00]	66.00 [56.00, 79.00]	<0.001
Mean BP, mmHg		81.00 [71.00, 93.00]	79.00 [69.00, 91.00]	80.00 [70.00, 92.00]	84.00 [73.00, 96.00]	83.00 [73.00, 93.00]	<0.001
SpO2, %		98.00 [95.00, 100.00]	99.00 [96.00, 100.00]	98.00 [95.00, 100.00]	98.00 [95.00, 100.00]	99.00 [96.00, 100.00]	<0.001
**Laboratory tests**							
Glucose, mg/dL		139.00 [107.00, 192.00]	140.00 [109.00, 197.00]	148.00 [114.00, 197.50]	141.00 [111.00, 192.50]	125.00 [102.00, 173.00]	<0.001
Hemoglobin, g/dL		10.70 [9.00, 12.60]	10.00 [8.60, 12.10]	10.50 [8.80, 12.40]	11.30 [9.40, 13.20]	11.10 [9.10, 12.70]	<0.001
Platelets, K/uL		189.00 [143.00, 244.00]	139.00 [111.75, 172.00]	176.00 [140.00, 224.00]	210.00 [169.00, 256.50]	235.00 [189.50, 295.00]	<0.001
White blood cells, K/uL		11.70 [8.70, 15.80]	16.85 [13.30, 20.92]	12.90 [10.20, 16.40]	10.90 [8.70, 13.30]	7.90 [6.30, 9.75]	<0.001
RDW, %		13.80 [13.00, 15.00]	13.60 [12.90, 14.93]	13.70 [12.90, 15.00]	13.80 [13.10, 15.00]	13.90 [13.10, 15.20]	0.051
Creatinine, mg/dL		1.10 [0.90, 1.70]	1.10 [0.90, 1.70]	1.10 [0.90, 1.70]	1.10 [0.80, 1.60]	1.10 [0.80, 1.70]	0.079
BUN, mg/dL		21.00 [15.00, 35.00]	21.00 [15.00, 35.00]	21.00 [16.00, 36.50]	21.00 [15.00, 34.50]	21.00 [14.00, 36.00]	0.364
Calcium, mg/dL		8.80 [8.30, 9.20]	8.80 [8.10, 9.20]	8.70 [8.30, 9.10]	8.80 [8.30, 9.10]	8.90 [8.40, 9.30]	0.007
Potassium, mEq/L		4.20 [3.90, 4.60]	4.20 [3.90, 4.50]	4.20 [3.90, 4.60]	4.20 [3.90, 4.60]	4.10 [3.80, 4.50]	0.018
Sodium, mEq/L		139.00 [136.00, 141.00]	139.00 [136.00, 141.00]	139.00 [136.00, 141.00]	138.00 [136.00, 141.00]	139.00 [136.00, 141.00]	0.467
Anion gap		15.00 [12.00, 18.00]	15.00 [12.00, 18.00]	15.00 [12.50, 18.00]	15.00 [13.00, 17.00]	14.00 [12.00, 17.00]	0.021
Bicarbonate, mEq/L		23.00 [20.00, 25.00]	22.50 [20.00, 25.00]	23.00 [20.00, 25.00]	23.00 [20.00, 24.00]	23.00 [21.00, 25.00]	<0.001
PWR		16.20 [11.27, 22.90]	8.82 [7.11, 10.10]	13.61 [12.46, 14.90]	19.17 [17.59, 21.10]	28.68 [25.41, 34.26]	<0.001
**Medications**							
Dobutamine use, n(%)		123 (5.2)	37 (6.2)	29 (4.9)	30 (5.0)	27 (4.5)	0.589
Dopamine use, n(%)		97 (4.1)	24 (4.0)	28 (4.7)	23 (3.9)	22 (3.7)	0.827
Milrinone use, n(%)		72 (3.0)	29 (4.9)	13 (2.2)	16 (2.7)	14 (2.4)	0.024
Epinephrine use, n(%)		297 (12.5)	110 (18.5)	58 (9.7)	59 (9.9)	70 (11.8)	<0.001
Norepinephrine use, n(%)		687 (28.9)	226 (37.9)	174 (29.2)	167 (28.1)	120 (20.2)	<0.001
Vasopressin use, n(%)		255 (10.7)	105 (17.6)	52 (8.7)	48 (8.1)	50 (8.4)	<0.001
Any vasoactive drug use, n(%)		1125 (47.2)	350 (58.7)	299 (50.3)	247 (41.5)	229 (38.5)	<0.001
Statin use, n(%)		1942 (81.6)	483 (81.0)	483 (81.2)	490 (82.4)	486 (81.7)	0.936
Aspirin use, n(%)		2003 (84.1)	512 (85.9)	513 (86.2)	504 (84.7)	474 (79.7)	0.006
Clopidogrel use, n(%)		506 (21.3)	104 (17.4)	135 (22.7)	152 (25.5)	115 (19.3)	0.003
**Comorbidities**							
Congestive heart failure, n(%)		1,262 (53.0)	290 (48.7)	315 (52.9)	340 (57.1)	317 (53.3)	0.035
Peripheral vascular disease, n(%)		353 (14.8)	81 (13.6)	97 (16.3)	85 (14.3)	90 (15.1)	0.588
Cerebrovascular disease, n(%)		344 (14.4)	87 (14.6)	71 (11.9)	86 (14.5)	100 (16.8)	0.125
Diabetes mellitus, n(%)		1082 (45.4)	246 (41.3)	273 (45.9)	280 (47.1)	283 (47.6)	0.115
COPD, n(%)		348 (14.6)	86 (14.4)	84 (14.1)	96 (16.1)	82 (13.8)	0.667
Hypertension, n(%)		794 (33.3)	224 (37.6)	190 (31.9)	189 (31.8)	191 (32.1)	0.092
Atrial fibrillation, n(%)		892 (37.5)	242 (40.6)	208 (35.0)	212 (35.6)	230 (38.7)	0.15
Hyperlipidemia, n(%)		1,464 (61.5)	366 (61.4)	369 (62.0)	364 (61.2)	365 (61.3)	0.992
End-stage renal disease, n(%)		167 (7.0)	34 (5.7)	37 (6.2)	42 (7.1)	54 (9.1)	0.11
Acute kidney failure, n(%)		1,035 (43.5)	281 (47.1)	268 (45.0)	258 (43.4)	228 (38.3)	0.016
Cardiogenic shock, n(%)		456 (19.2)	120 (20.1)	120 (20.2)	122 (20.5)	94 (15.8)	0.122
Sepsis (sepsis3), n(%)		1,021 (42.9)	304 (51.0)	267 (44.9)	233 (39.2)	217 (36.5)	<0.001
**Interventions**							
Ventilation, n(%)		180 (7.6)	53 (8.9)	49 (8.2)	40 (6.7)	38 (6.4)	0.299
CRRT, n(%)		158 (6.6)	55 (9.2)	34 (5.7)	31 (5.2)	38 (6.4)	0.026
**Severity scores**							
SOFA score		2.00 [0.00, 4.00]	3.00 [1.00, 5.00]	1.00 [0.00, 3.00]	1.00 [0.00, 3.00]	1.00 [0.00, 3.00]	<0.001
APS III score		40.00 [30.00, 54.00]	44.00 [32.00, 61.25]	40.00 [30.00, 57.00]	39.00 [28.00, 51.00]	38.00 [29.00, 52.00]	<0.001
OASIS score		32.00 [26.00, 38.00]	34.00 [28.00, 40.00]	33.00 [27.00, 38.50]	31.00 [25.00, 37.00]	30.00 [25.00, 37.00]	<0.001
SAPS II score		37.00 [30.00, 46.00]	40.00 [32.75, 50.00]	37.00 [30.00, 46.00]	36.00 [28.50, 45.00]	35.00 [28.00, 42.00]	<0.001
**Events**							
ICU mortality, n(%)		237 (10.0)	85 (14.3)	64 (10.8)	47 (7.9)	41 (6.9)	<0.001
Hospital mortality, n(%)		316 (13.3)	103 (17.3)	86 (14.5)	73 (12.3)	54 (9.1)	<0.001

BP, blood pressure; SpO2, peripheral capillary oxygen saturation; RDW, red cell distribution width; BUN, blood urea nitrogen; PWR, platelet-to-white blood cell ratio; COPD, chronic obstructive pulmonary disease; CRRT, continuous renal replacement therapy; SOFA, sequential organ failure assessment; APS III, acute physiology score III; OASIS, Oxford Acute Severity of Illness Score; SAPS II, Simplified Acute Physiology Score II; ICU, intensive care unit.

a Continuous data are presented as median (interquartile range), and categorical data as frequencies (percentages).

To validate the generalizability of the findings from the primary cohort, we additionally included 7,054 patients from the eICU-CRD database as one external validation cohort. In this cohort, the median age was 67.00 (57.00, 77.00) years, 4,405 (62.4%) were male, in-hospital mortality was 8.9% (630 cases), ICU mortality was 5.8% (406 cases), and the median PWR was 19.39 (14.34, 25.83). Consistent with the primary cohort, in the eICU cohort, higher PWR was significantly associated with lower proportions of vasoactive drug use, lower WBC counts, lower APS III and SOFA scores, as well as lower in-hospital and ICU mortality rates (all *P* < 0.001) (Supplementary Table 1). In addition, we included 252 patients from Guangdong Provincial Hospital of Traditional Chinese Medicine as another validation cohort. The mean age was 63.62 ± 12.81 years, 195 (77.4%) were male, the median PWR was 23.24 (18.02, 27.70), and in-hospital mortality was 7.9% (20 cases). Consistent with the primary cohort, higher PWR was significantly associated with lower in-hospital mortality (*P* = 0.002) (Supplementary Table 2).

### 3.2 Association between PWR and in-hospital/ICU mortality in the derivation cohort

We first used RCS to analyze the potential nonlinear association between PWR and the primary outcomes. Figure 2 displays the RCS analyses adjusted for age and sex. In the MIMIC-IV cohort, extreme PWR values (<6.77 or >36.32) were trimmed (238 cases excluded, leaving 2,143 for analysis); in the eICU-CRD cohort, values <8.31 or >38.96 were trimmed (706 cases excluded, leaving 6,348 for analysis). The results showed no significant nonlinear relationship between PWR and in-hospital or ICU mortality. Specifically, the *P* for nonlinearity for in-hospital mortality was 0.686 in MIMIC-IV and 0.293 in eICU-CRD; for ICU mortality, the corresponding values were 0.731 and 0.415, respectively. For all other analyses in this study, the full analytic sample was used. Kaplan–Meier survival curves (Figure 3) demonstrated that with increasing PWR, both in-hospital and ICU mortality decreased in the MIMIC-IV cohort (hospital mortality, *P* = 0.001; ICU mortality, *P* = 0.016); in the eICU-CRD cohort, these decreasing trends were even more pronounced (hospital mortality, *P* < 0.0001; ICU mortality, *P* < 0.0001).

**Figure 2:**
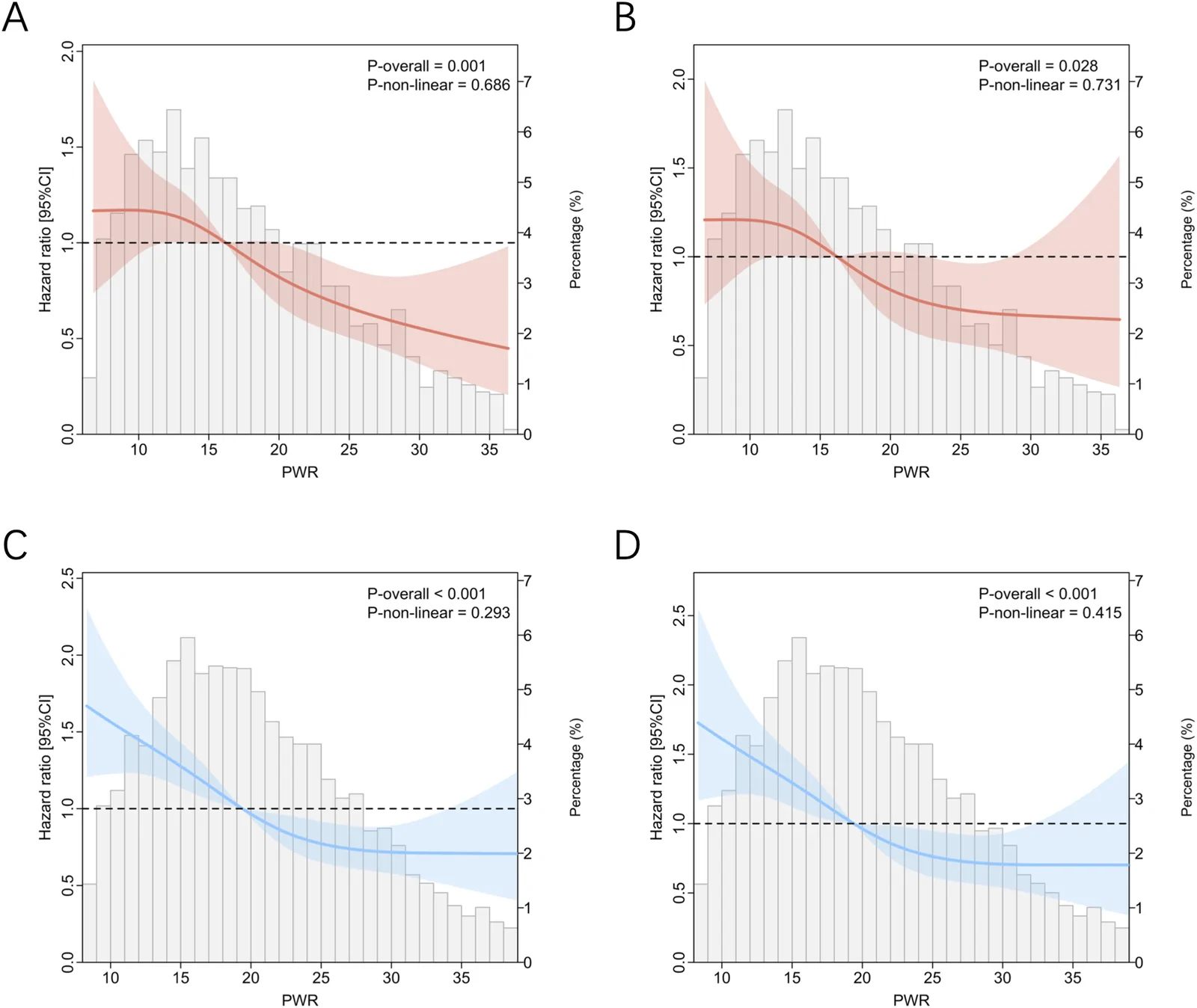
Restricted cubic spline analyses of the association between PWR and in-hospital/ICU mortality in AMI patients, adjusted for age and sex. **(A)** In-hospital mortality in the MIMIC-IV cohort; **(B)** ICU mortality in the MIMIC-IV cohort; **(C)** In-hospital mortality in the eICU-CRD cohort; **(D)** ICU mortality in the eICU-CRD cohort.

**Figure 3:**
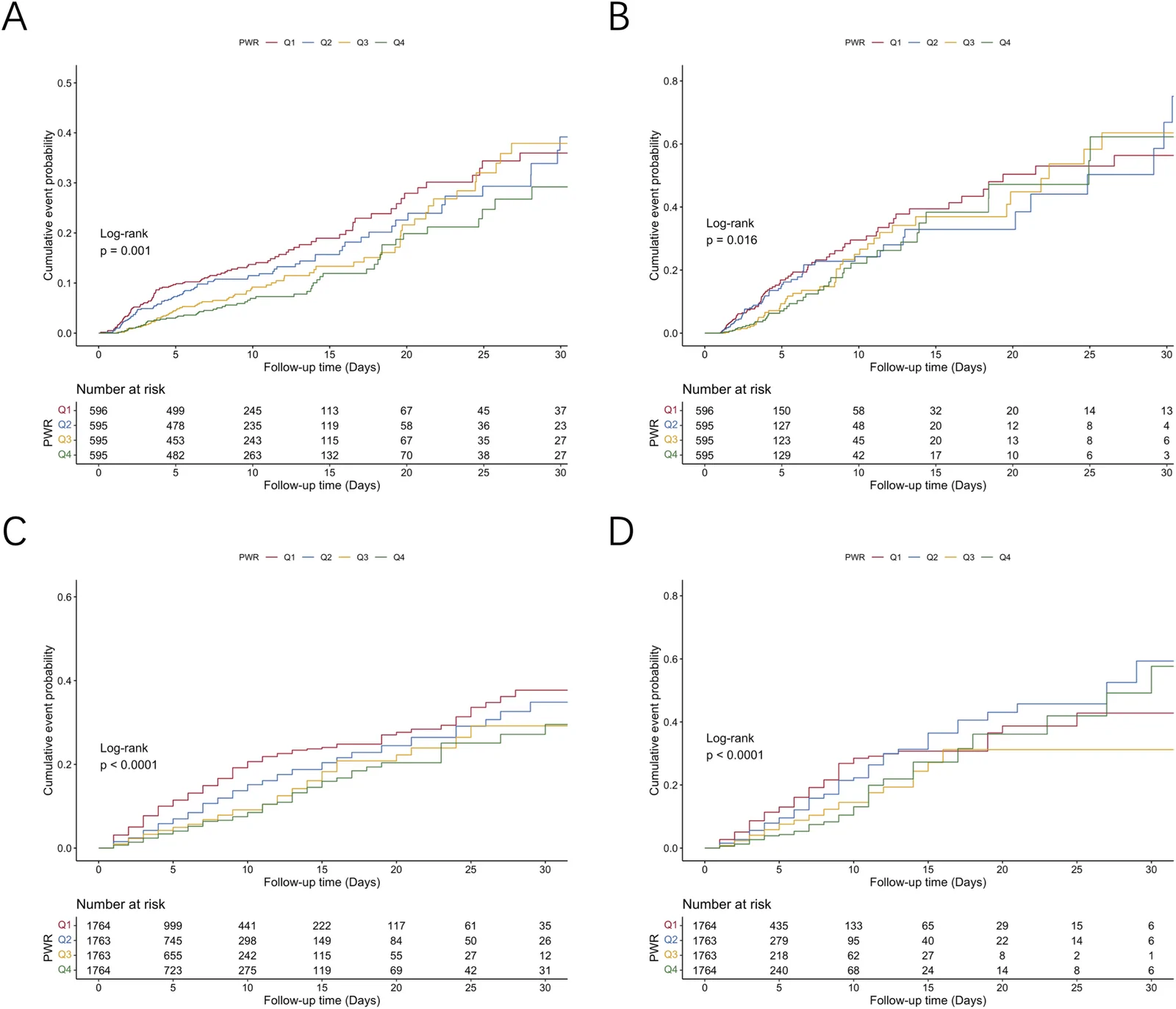
Kaplan–meier survival curves for in-hospital and ICU mortality stratified by PWR quartiles in AMI patients. **(A)** In-hospital mortality in the MIMIC-IV cohort; **(B)** ICU mortality in the MIMIC-IV cohort; **(C)** In-hospital mortality in the eICU-CRD cohort; **(D)** ICU mortality in the eICU-CRD cohort.

We then used multivariable logistic regression models to evaluate the independent association between PWR and clinical outcomes in the primary cohort. As shown in Table 2, after adjustment for age and sex (Model 2), continuous PWR was significantly negatively associated with both in-hospital mortality (OR = 0.968, 95% CI: 0.954–0.982, *P* < 0.001) and ICU mortality (OR = 0.966, 95% CI: 0.949–0.982, *P* < 0.001). After adjustment for all prespecified covariates in Model 3, including medication history, laboratory parameters, comorbidities, and disease severity scores, the inverse associations between PWR and short-term mortality remained significant. Specifically, each 1-unit increase in PWR was associated with lower in-hospital mortality (OR = 0.976, 95% CI 0.960–0.991, *P* = 0.003) and lower ICU mortality (OR = 0.977, 95% CI 0.959–0.994, *P* = 0.012). When expressed per IQR (11.63), each 1-IQR increase in PWR remained significantly associated with a lower risk of in-hospital mortality (OR = 0.754, 95% CI 0.622–0.900, *P* = 0.003), corresponding to a 24.6% relative risk reduction, and with a lower risk of ICU mortality (OR = 0.763, 95% CI 0.614–0.932, *P* = 0.012), corresponding to a 23.7% relative risk reduction (Supplementary Table 3). When PWR was categorized by quartiles, using the lowest quartile (Q1) as reference, the Q4 group had significantly lower in-hospital mortality (OR = 0.568, 95% CI: 0.374–0.856, *P* = 0.007) and ICU mortality (OR = 0.575, 95% CI: 0.359–0.909, *P* = 0.019) compared with Q1. Trend tests indicated a significant dose-response decrease in both in-hospital and ICU mortality with rising PWR levels (*P* for trend = 0.006 and 0.008, respectively).

**Table 2:** The association between the PWR and in-hospital mortality and ICU mortality in patients with AMI from MIMI-IV.

Variables	Model 1		Model 2		Model 3	
	OR (95% CI)	*P* value	OR (95% CI)	*P* value	OR (95% CI)	*P* value
**Hospital mortality**						
Continuous variable per unit	0.969 (0.954, 0.982)	<0.001	0.968 (0.954, 0.982)	<0.001	0.976 (0.96, 0.991)	0.00295
Q1 (Ref)	Reference		Reference		Reference	
Q2	0.809 (0.591, 1.104)	0.18218	0.780 (0.568, 1.068)	0.12231	0.901 (0.619, 1.311)	0.58531
Q3	0.669 (0.483, 0.924)	0.01521	0.637 (0.457, 0.882)	0.00703	0.785 (0.532, 1.156)	0.22141
Q4	0.478 (0.334, 0.676)	<0.001	0.466 (0.325, 0.663)	<0.001	0.568 (0.374, 0.856)	0.0073
*P* for trend		<0.001		<0.001		0.00647
**ICU mortality**						
Continuous variable per unit	0.966 (0.949, 0.981)	<0.001	0.966 (0.949, 0.982)	<0.001	0.977 (0.959, 0.994)	0.01166
Q1 (Ref)	Reference		Reference		Reference	
Q2	0.725 (0.511, 1.023)	0.0683	0.708 (0.499, 1.002)	0.0522	0.832 (0.547, 1.264)	0.389
Q3	0.516 (0.352, 0.748)	<0.001	0.500 (0.340, 0.726)	<0.001	0.618 (0.393, 0.966)	0.03586
Q4	0.445 (0.298, 0.654)	<0.001	0.441 (0.294, 0.65)	<0.001	0.575 (0.359, 0.909)	0.01916
*P* for trend		<0.001		<0.001		0.00801

Model 1 was unadjusted.

Model 2 was adjusted by Age, Gender.

Model 3 was adjusted by Age, Gender, Statin use, Aspirin use, Clopidogrel use, Creatinine, Hemoglobin, Calcium, Potassium, Congestive heart failure, Peripheral vascular disease, Cerebrovascular disease, Diabetes mellitus, Hypertension, Atrial fibrillation, Hyperlipidemia, COPD, CRRT, SOFA score, APS III score.

To assess whether significant multicollinearity existed in the logistic regression models, we calculated Spearman's rank correlation coefficients and the square root of the VIF (Supplementary Table 4). Spearman's correlation analysis showed that all absolute correlation coefficients between continuous variables were <0.6, indicating no strong correlations. The VIF diagnosis revealed that the square root of VIF for all variables was <2. Collectively, these results indicate no significant multicollinearity in the logistic models, ensuring the stability and reliability of the estimates.

### 3.3 Subgroup analysis

Subgroup analyses evaluated whether the association between PWR and the primary outcomes differed across demographic and clinical strata, including sex, age, and comorbidities (hypertension, diabetes, congestive heart failure, atrial fibrillation, COPD, end-stage renal disease, acute kidney injury, and sepsis). As shown in the forest plot (Figure 4), subgroup analyses revealed no significant interactions between PWR and any of the prespecified subgroup variables (all FDR-adjusted *P* values > 0.05 after Benjamini-Hochberg correction). For in-hospital mortality, the diabetes-PWR interaction was statistically significant before FDR correction (*P* = 0.032) but was no longer significant after correction (FDR = 0.316). For ICU mortality, the diabetes-PWR interaction was likewise significant before FDR correction (*P* = 0.009) but not after correction (FDR = 0.087). The FDR-adjusted *P* values for interactions with all other subgroup variables were > 0.05. Overall, the inverse association between PWR and short-term mortality was consistent in direction across the vast majority of prespecified subgroups, indicating that the findings were robust.

**Figure 4:**
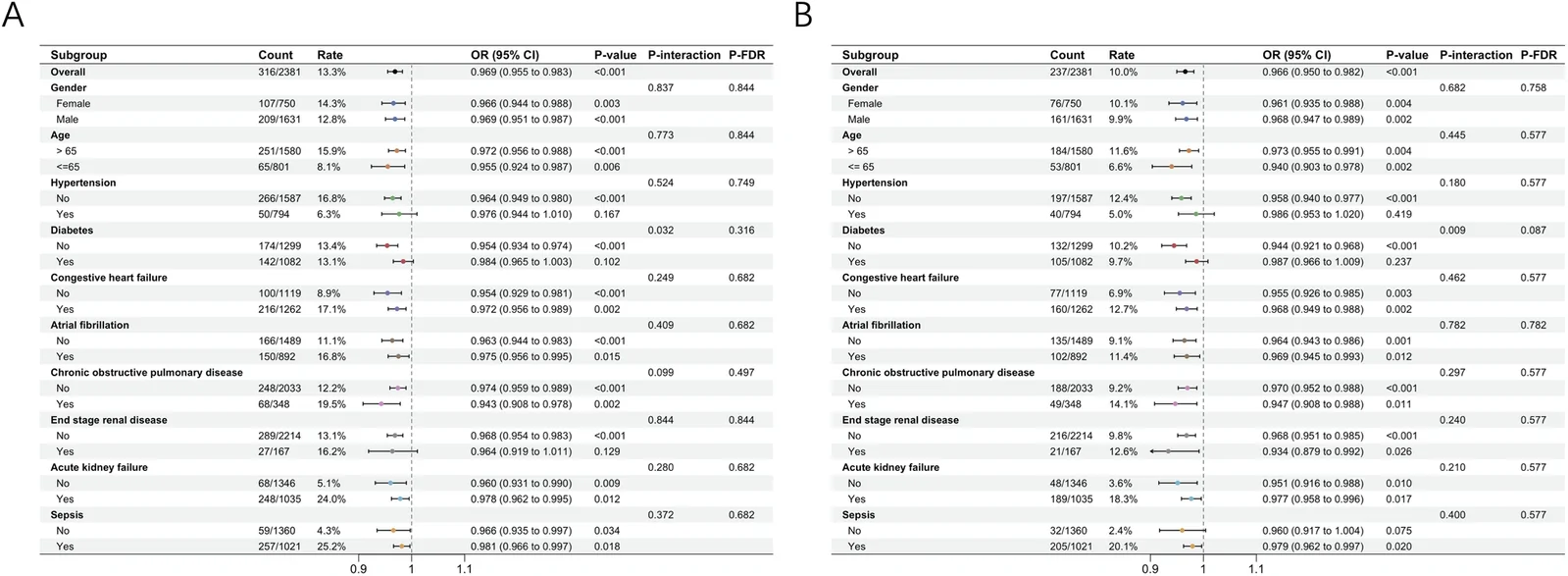
Subgroup analyses for the association between PWR and in-hospital/ICU mortality in the MIMIC-IV cohort. **(A)** In-hospital mortality; **(B)** ICU mortality.

### 3.4 Sensitivity analysis

To validate the association between PWR and short-term prognosis, we first performed sensitivity analyses using Cox proportional hazards regression (Supplementary Table 5). After full adjustment, each unit increase in PWR was significantly associated with reduced in-hospital mortality (HR = 0.978, 95% CI: 0.965–0.992, *P* = 0.001) and ICU mortality (HR = 0.984, 95% CI: 0.969–0.999, *P* = 0.039), suggesting that higher PWR is an independent protective factor for short-term mortality in AMI patients. Quartile analyses demonstrated a significant graded association across all three outcomes, with mortality gradually decreasing from the lowest (Q1) to the highest (Q4) PWR quartile (all *P* for trend < 0.05). Furthermore, we explored the association between PWR and long-term mortality. However, after full adjustment (Model 3), each unit increase in PWR was not significantly associated with 180-day mortality (HR = 0.992, 95% CI: 0.983–1.001, *P* = 0.078) or 365-day mortality (HR = 0.994, 95% CI: 0.986–1.002, *P* = 0.163) (Supplementary Table 6).

To further account for competing risks, we performed sensitivity analyses using Fine-Gray subdistribution hazard models (Supplementary Table 7). After multivariable adjustment (Model 3), PWR remained significantly associated with a reduced cumulative incidence of in-hospital mortality (sHR = 0.976, 95% CI: 0.960–0.991, *P* = 0.002), and the results were consistent for ICU mortality (sHR = 0.974, 95% CI: 0.955–0.993, *P* = 0.007). These findings indicate that the negative association between PWR and short-term mortality remained robust after controlling for competing events. Collectively, both sensitivity analyses yielded results consistent with the primary analysis, confirming the robustness of PWR as an independent prognostic indicator for short-term outcomes in AMI patients.

### 3.5 Calibration and incremental prediction

The calibration curve of PWR showed close agreement between the mean predicted and observed risks, indicating acceptable overall calibration (Supplementary Figure 1, Supplementary Table 8). To assess the incremental predictive value of PWR, we constructed a base model using the covariates in Model 3 and then added PWR to create a PWR-enhanced model. Decision curve analysis (DCA) was performed for both outcomes—in-hospital mortality and ICU mortality—to assess the incremental clinical utility of PWR (Supplementary Figure 1, Supplementary Table 9). In the in-hospital mortality analysis, the PWR-enhanced model showed a higher net benefit than the base model across a threshold probability range of approximately 0.20–0.60. At a threshold probability of 0.35, the difference in normalized net benefit between the PWR-enhanced and base models was statistically significant (difference = 0.00489, 95% CI: 0.00065–0.00908). For ICU mortality, the PWR-enhanced model had significantly higher net benefit than the base model at threshold probabilities of 0.15 and 0.35 (0.15: difference = 0.00426, 95% CI: 0.00086–0.00791; 0.35: difference = 0.00283, 95% CI: 0.00003–0.00578), and its curve remained consistently above that of the base model across the 0.15–0.40 threshold range. These findings suggest that incorporating PWR into the base risk model improves net benefit at some clinically relevant thresholds, supporting its potential value as an adjunctive prognostic marker with incremental predictive value.

### 3.6 Validation analysis

To assess the generalizability of our findings, we performed external validation in the eICU-CRD database (7,054 AMI patients) and internal validation in a single-center cohort of 252 AMI patients admitted to the cardiovascular ward. The results are presented in Table 3. In the eICU validation cohort, multivariable logistic regression showed that, using the lowest PWR quartile (Q1) as reference, the two highest quartiles (Q3 and Q4) had significantly lower in-hospital mortality (Q3: OR = 0.659, 95% CI: 0.504–0.858, *P* = 0.002; Q4: OR = 0.596, 95% CI: 0.452–0.781, *P* < 0.001) and ICU mortality (Q3: OR = 0.641, 95% CI: 0.460–0.885, *P* = 0.008; Q4: OR = 0.489, 95% CI: 0.341–0.692, *P* < 0.001). However, no significant differences were observed for the Q2 group in either outcome (in-hospital: *P* = 0.305; ICU: *P* = 0.901). Trend tests demonstrated a significant dose-response relationship between higher PWR levels and reduced in-hospital and ICU mortality (both *P* for trend < 0.001). When PWR was analyzed as a continuous variable, the negative association remained significant; each unit increase in PWR was associated with lower in-hospital mortality (OR = 0.986, 95% CI: 0.976–0.995, *P* = 0.005) and lower ICU mortality (OR = 0.980, 95% CI: 0.968–0.992, *P* = 0.001). In the eICU-CRD external validation cohort, each 1-IQR (11.49) increase in PWR was significantly associated with a lower risk of in-hospital mortality (OR = 0.850, 95% CI 0.756–0.944, *P* = 0.005), corresponding to a 15.0% relative reduction in risk. Each 1-IQR increase in PWR was also significantly associated with a lower risk of ICU mortality (OR = 0.793, 95% CI 0.688–0.912, *P* = 0.001), corresponding to a 20.7% relative reduction in risk. These findings were consistent in direction with those observed in the derivation cohort (Supplementary Table 3). Consistent with the above, in the internal validation cohort, after stepwise adjustment for confounders, patients in the Q4 group had significantly lower in-hospital mortality than those in the Q1 group (Model 3: OR = 0.082, 95% CI: 0.007–0.552, *P* = 0.020), with a significant decreasing trend from Q1 to Q4 (*P* for trend = 0.010). Results remained consistent when PWR was entered as a continuous variable (OR = 0.874, 95% CI: 0.788–0.950, *P* = 0.004), indicating that each unit increase in PWR was associated with approximately a 12.6% reduction in in-hospital mortality.

**Table 3:** External and internal validation of the association of PWR with in-hospital mortality and ICU mortality.

Cohort/Outcome	Variables	Model 1		Model 2		Model 3	
		OR(95% CI)	*P* value	OR(95% CI)	*P* value	OR(95% CI)	*P* value
eICU-CRD Cohort							
Hospital mortality	Continuous variable per unit	0.954 (0.944–0.964)	<0.001	0.957 (0.947–0.967)	<0.001	0.986 (0.976–0.995)	0.00453
	Q1 (Ref)	Reference		Reference		Reference	
	Q2	0.526 (0.426–0.647)	<0.001	0.572 (0.462–0.706)	<0.001	0.884 (0.698–1.118)	0.3046
	Q3	0.333 (0.261–0.42)	<0.001	0.358 (0.280–0.454)	<0.001	0.659 (0.504–0.858)	0.00216
	Q4	0.315 (0.246–0.400)	<0.001	0.326 (0.254–0.416)	<0.001	0.596 (0.452–0.781)	<0.001
	*P* for trend		<0.001		<0.001		<0.001
ICU mortality	Continuous variable per unit	0.943 (0.931–0.956)	<0.001	0.947 (0.934–0.959)	<0.001	0.980 (0.968–0.992)	0.00149
	Q1 (Ref)	Reference		Reference		Reference	
	Q2	0.562 (0.438–0.718)	<0.001	0.604 (0.470–0.773)	<0.001	0.983 (0.745–1.293)	0.90144
	Q3	0.311 (0.230–0.416)	<0.001	0.332 (0.245–0.445)	<0.001	0.641 (0.460–0.885)	0.00763
	Q4	0.249 (0.179–0.340)	<0.001	0.260 (0.186–0.356)	<0.001	0.489 (0.341–0.692)	<0.001
	*P* for trend		<0.001		<0.001		<0.001
Local Cohort							
Hospital mortality	Continuous variable per unit	0.887 (0.825–0.946)	0.00053	0.884 (0.819–0.945)	0.00061	0.874 (0.788–0.950)	0.00423
	Q1 (Ref)	Reference		Reference		Reference	
	Q2	0.288 (0.077–0.885)	0.04076	0.283 (0.073–0.900)	0.04379	0.162 (0.027–0.751)	0.0285
	Q3	0.139 (0.021–0.541)	0.01227	0.134 (0.020–0.539)	0.01232	0.130 (0.016–0.685)	0.02794
	Q4	0.139 (0.021–0.541)	0.01227	0.140 (0.021–0.564)	0.01432	0.082 (0.007–0.552)	0.01964
	*P* for trend		0.00193		0.00236		0.00959

Model 1 was unadjusted.

Model 2 was adjusted by Age, Gender.

Model 3 was adjusted by Age, Gender, Statin use, Aspirin use, Clopidogrel use, Creatinine, Hemoglobin, Calcium, Potassium, Congestive heart failure, Peripheral vascular disease, Cerebrovascular disease, Diabetes mellitus, Hypertension, Atrial fibrillation, Hyperlipidemia, COPD, CRRT, SOFA score, APS III score.

In addition, E-value analysis was performed to assess the potential impact of unmeasured confounding on the robustness of the results (Supplementary Table 10). In the MIMIC-IV cohort, the E-value (the minimum risk ratio that an unmeasured confounder would need to have with both exposure and outcome to fully explain away the observed association) for the inverse association between PWR and in-hospital mortality was 2.92 (conservative estimate: 1.61); for ICU mortality, the corresponding E-value was 2.87 (conservative estimate: 1.43). In the eICU validation cohort, the E-value for the inverse association between PWR and in-hospital mortality was 2.74 (conservative estimate: 1.88); for ICU mortality, the corresponding E-value was 3.51 (conservative estimate: 2.25). These E-value estimates are substantially larger than typical effect sizes of common confounders, suggesting that unmeasured confounding is unlikely to fully account for the observed negative association between PWR and short-term mortality, further supporting the robustness of our conclusions.

### 3.7 Mediation analysis

To assess whether SAPS II score and vasoactive drug use mediated the association between PWR and ICU mortality, we performed a mediation analysis with both as mediators, adjusting for the same covariates as in Model 3 of the primary analysis. The results are shown in Table 4. Both SAPS II and vasoactive drug use significantly mediated the association between PWR and ICU mortality. For SAPS II, the average causal mediation effect (ACME) was −0.000842 (95% CI: −0.001680, −0.000288, *P* < 0.001), the average direct effect (ADE) was −0.002408 (95% CI: −0.004515, −0.000723, *P* = 0.002), the total effect was −0.003250 (95% CI: −0.005723, −0.001370, *P* < 0.001), and the proportion mediated was 25.9% (95% CI: 10.6% to 52.6%, *P* < 0.001). For vasoactive drug use, the ACME was −0.000329 (95% CI: −0.000596, −0.000141, *P* < 0.001), the ADE was −0.001440 (95% CI: −0.002413, −0.000403, *P* = 0.012), the total effect was −0.001769 (95% CI: −0.002800, −0.000788, *P* < 0.001), and the proportion mediated was 18.6% (95% CI: 8.4% to 47.0%, *P* < 0.001). These findings indicate that SAPS II and vasoactive drug use explained 25.9% and 18.6% of the total effect of PWR, respectively, suggesting that the protective effect of PWR operates at least through these two pathways. The remaining direct effect implies that other potential mechanisms (e.g., anti-inflammatory, endothelial protection) warrant further investigation.

**Table 4:** Mediation analysis of SAPS II score and vasoactive drug use on the association between PWR and ICU mortality.

Effect	Estimate	95% CI	*p*-value
**SAPS II score**			
ACME	−0.000842	−0.001680 to −0.000288	<0.001
ADE	−0.002408	−0.004515 to −0.000723	0.002
Total Effect	−0.003250	−0.005723 to −0.001370	<0.001
Proportion Mediated (%)	25.91	10.58 to 52.63	<0.001
**Any vasoactive drug use**			
ACME	−0.000329	−0.000596 to −0.000141	<0.001
ADE	−0.001440	−0.002413 to −0.000403	0.012
Total Effect	−0.001769	−0.002800 to −0.000788	<0.001
Proportion Mediated (%)	18.60	8.41 to 46.99	<0.001

Adjusted for the same covariates as in Model 3 of the primary analysis.

SAPS II, Simplified Acute Physiology Score II; ACME, average causal mediation effect; ADE, Average direct effect; CI, Confidence interval.

## 4 Discussion

In this study, we systematically evaluated the prognostic value of the platelet-to-white blood cell ratio (PWR) for short-term outcomes in patients with acute myocardial infarction (AMI), using a retrospective analysis of the primary MIMIC-IV cohort and external validation in both the eICU-CRD database and an independent single-center cohort. We found that PWR measured within the first 24 h of admission was significantly and negatively associated with in-hospital and intensive care unit (ICU) mortality, and these associations persisted after adjusting for a wide range of potential confounders. Consistent findings were observed across all validation cohorts.

The adverse prognosis following AMI is fundamentally driven by excessive inflammation and a prothrombotic state (21), and thrombo-inflammation has been shown to independently correlate with post-AMI outcomes (22, 23). In recent years, with growing recognition of the roles of inflammation, thrombosis, and metabolism in cardiovascular disease, a series of composite inflammatory markers derived from routine blood counts have been proposed and validated for prognostic value. The neutrophil-to-lymphocyte ratio (NLR), as a quantifiable indicator of systemic inflammation, reflects the imbalance between neutrophil-mediated pro-inflammatory status and lymphocyte-mediated immunoregulation, and has been consistently associated with in-hospital mortality in AMI patients (24–26). Similarly, the platelet-to-lymphocyte ratio (PLR) has also been linked to prognosis in AMI (27, 28). However, studies have shown that preoperative low PWR is an independent predictor of postoperative acute kidney injury, with predictive performance superior to that of NLR and the systemic immune-inflammation index (SII) (29). In a prospective cohort of 1,453 patients with acute decompensated heart failure (ADHF), Huang et al. demonstrated that PWR was negatively associated with 30-day all-cause mortality and provided better short-term mortality prediction than the traditional heart failure marker NT- proBNP (15). Furthermore, a large study encompassing patients with COVID-19, acute heart failure, myocardial infarction, and stroke confirmed that PWR exhibited a stronger association with mortality than NLR and PLR (12). Thus, exploring the predictive value of PWR for short-term prognosis in AMI is of significant clinical importance for optimizing current risk stratification and guiding individualized management.

The prognostic significance of PWR observed in our study can be deeply understood through the intertwined dual pathophysiology of inflammation and thrombosis during AMI development. AMI is essentially an acute coronary occlusion caused by rupture or erosion of an atherosclerotic plaque (30, 31), leading to severe ischemia and hypoxia in the corresponding myocardial region and ultimately irreversible myocyte necrosis (32, 33), and is characterized by a robust local and systemic inflammatory response (34). Recent research has established that atherosclerosis is not merely a lipid storage disease but a chronic inflammatory condition, and AMI represents an acute exacerbation of this chronic process (35–37). Moreover, inflammation is considered a core pathological mechanism underlying myocardial ischemia-reperfusion injury and tissue necrosis, and modulating inflammatory pathways has become an important strategy to mitigate myocardial damage and improve prognosis (38). Blood cells, including white blood cells (WBC) and platelets, are intimately involved in atherogenesis and myocardial infarction as part of the inflammatory response (39). Leukocytes are major mediators of inflammation, playing a critical role in host defense, and have been identified as independent predictors of all-cause mortality (40). As core effector cells of inflammation, leukocytes are massively recruited to the injured vascular site after plaque rupture, releasing various pro-inflammatory cytokines, matrix metalloproteinases, and oxidative stress products, which not only exacerbate myocardial tissue injury but also impair functional recovery through endothelial dysfunction, microvascular obstruction, and aggravated reperfusion injury, ultimately affecting patient outcomes (41, 42). Meanwhile, the role of platelets in the pathogenesis of myocardial infarction extends far beyond classical hemostasis and thrombosis (43). Platelets, produced by megakaryocytes through a complex process, promote the differentiation of monocytes and macrophages toward a pro-atherosclerotic phenotype and form monocyte-platelet aggregates (MPA) with monocytes, thereby linking inflammation and thrombosis (44). Furthermore, platelets serve as a key bridge between inflammation and thrombosis; activated platelets directly mediate leukocyte recruitment and rolling adhesion to the vascular endothelium via surface P-selectin binding to P-selectin glycoprotein ligand-1 (PSGL-1) on leukocytes (45–47). As leukocytes progressively accumulate and platelets are increasingly consumed, leukocyte-platelet aggregates (LPAs) form, further amplifying inflammation and leading to adverse outcomes (48–50). In addition to P-selectin, activated platelets release various inflammatory mediators such as CD40 ligand (CD40L), platelet factor 4 (PF4), and RANTES, which further amplify the local and systemic inflammatory cascade, exacerbating myocardial injury and maladaptive remodeling (51, 52). This bidirectional interaction between platelets and leukocytes creates a vicious cycle—inflammation activates platelets, and activated platelets in turn aggravate inflammation—collectively driving the progression of coronary atherosclerosis and acute thrombotic events. Studies have found that a low PWR may reflect a deranged balance between platelets and leukocytes; lower PWR indicates persistently low platelet counts and persistently elevated leukocyte counts, suggesting ongoing systemic inflammation (12). It is also worth noting that a lower PWR may reflect thrombocytopenia. Thrombocytopenia increases bleeding risk and can lead to bleeding events (53, 54), particularly in patients with AMI receiving dual antiplatelet therapy and anticoagulation (55). However, the association between PWR and bleeding risk may not operate in a single direction; rather, it may represent the net result of two opposing mechanisms. A lower PWR may increase bleeding propensity through thrombocytopenia while also contributing to greater disease severity through a higher inflammatory burden. These two processes may coexist and interact (56), rendering the association between a lower PWR and bleeding risk nonspecific. In contrast, during healthy recovery from inflammation, leukocyte and platelet populations exhibit coordinated regulation, with leukocyte counts declining exponentially while platelet counts increase linearly (57). Thus, an elevated PWR can be viewed as a marker of favorable dynamic balance, possibly indicating that deleterious platelet-leukocyte crosstalk has been effectively interrupted, and thus closely correlates with a lower risk of adverse cardiovascular events.

In this context, PWR, as a composite indicator integrating platelet count (reflecting thrombotic tendency) and leukocyte count (reflecting inflammatory burden), has unique value in simultaneously capturing both core pathological elements. Studies have shown that STEMI patients with a high PWR on admission exhibit higher rates of spontaneous reperfusion and lower incidence of no-reflow phenomenon, consequently reducing severe complications and improving short-term prognosis (14). Consistent with our findings, higher PWR values were associated with lower mortality risk in AMI patients, suggesting PWR may serve as a simple marker for risk assessment and clinical decision-making. In our study, after comprehensive adjustment for confounders (Model 3), PWR was significantly and negatively associated with both in-hospital mortality (OR = 0.976, 95% CI: 0.960–0.991) and ICU mortality (OR = 0.977, 95% CI: 0.959–0.994). This protective association remained consistent across both internal and external validation cohorts, and was robust after controlling for competing risks and unmeasured confounding. In the subgroup analyses, to account for multiple comparisons, we applied the Benjamini–Hochberg false discovery rate (FDR) method separately to interaction tests for hospital mortality and ICU mortality. After FDR correction, none of the prespecified subgroup interactions remained statistically significant (all *P*-FDR > 0.05). Accordingly, we did not identify any prespecified subgroup factor as a significant effect modifier of the association between PWR and short-term mortality. Additionally, mediation analysis suggested that disease severity, hemodynamic instability, and circulatory failure may serve as important intermediate pathways through which PWR influences prognosis. However, given the uncertainty in temporal ordering caused by overlapping measurement windows for the exposure and mediator, as well as the lack of key molecular markers (e.g., specific inflammatory cytokines) in the database, we were unable to directly validate the mediating pathways at the molecular level. Accordingly, the mediation results should be regarded as exploratory and hypothesis-generating only. Furthermore, studies have proposed that inflammatory heterogeneity after PCI comprises two dimensions: an acute inflammatory phenotype (elevated WBC/CRP/NLR) and a chronic low-grade inflammatory phenotype (elevated ESR) (58). PWR primarily reflects WBC-associated acute inflammation and, from a clinical perspective, cannot yet fully replace the inflammatory phenotypes captured by other markers. Nevertheless, as a simple, single-dimensional index, it merits future analysis alongside ESR, CRP, and NLR within a broader framework of inflammatory heterogeneity and precision risk stratification.

Collectively, our results indicate that PWR may have predictive value for early mortality risk in AMI patients by comprehensively reflecting the balance between systemic inflammatory burden and platelet activation status. As a composite marker integrating inflammation and thrombotic load, PWR can be rapidly obtained from routine blood tests with no additional cost, offering excellent accessibility and clinical applicability, and has demonstrated stable and superior prognostic capability across multiple disease spectra (59–63). Therefore, clinicians should pay attention to this easily obtainable hematological parameter upon AMI admission and incorporate it into early risk assessment systems to help identify high-risk individuals and guide timely treatment adjustments. For high-risk patients with markedly reduced PWR, intensified monitoring and early individualized therapy may further reduce short-term mortality and improve overall clinical outcomes.

Several limitations of this study should be acknowledged. First, this is a retrospective observational study, subject to inherent selection and information biases that may partly affect the results. Second, PWR was measured only at the first recording within 24 h of admission, without dynamic monitoring during hospitalization or follow-up; thus, we could not fully capture its fluctuation patterns during the acute and recovery phases of AMI, nor their impact on outcomes. Third, in the internal validation cohort, patients were admitted directly to the emergency department or cardiology department after AMI onset, so only in-hospital all-cause mortality could be extracted, and ICU mortality could not be independently calculated; therefore, outcome analyses in this cohort were limited to in-hospital mortality. Fourth, due to the inherent design of the databases, we were unable to obtain detailed pre-admission medication histories, symptom-to-reperfusion times, or specific procedural details of coronary angiography and percutaneous coronary intervention (PCI) (such as infarct-related artery, TIMI flow grade, whether stenting was performed, stent type and number). Future studies should therefore implement multi-center, large-scale prospective cohorts with more comprehensive data collection, and incorporate advanced big-data analytics and machine learning approaches to construct and validate prediction models that include multiple risk factors including PWR, and to further explore the interactions between PWR and other modifiable factors during the acute phase of AMI.

## 5 Conclusions

This study found that the platelet-to-white blood cell ratio (PWR) was significantly negatively correlated with in-hospital and ICU all-cause mortality in patients with acute myocardial infarction (AMI). This finding suggests that PWR may have potential as a simple and low-cost adjunctive indicator for early risk stratification in preliminary screening, and may provide additional reference information for identifying high-risk patients and optimizing the timing of subsequent interventions in clinical practice. Nonetheless, these conclusions require further validation in larger, prospective studies.

## Data Availability

Publicly available datasets were analyzed in this study. This data can be found here: The MIMIC-IV database and the eICU-CRD database. The links are presented in the manuscript in the form of references.
